# Period Family of Clock Genes as Novel Predictors of Survival in Human Cancer: A Systematic Review and Meta-Analysis

**DOI:** 10.1155/2020/6486238

**Published:** 2020-08-10

**Authors:** Fan Deng, Kai Yang, Gang Zheng

**Affiliations:** ^1^Department of Oral and Maxillofacial Surgery, The First Affiliated Hospital of Chongqing Medical University, 1 Youyi Road, Yuzhong District, Chongqing 400016, China; ^2^Anorectal Department, Chongqing Traditional Chinese Medicine Hospital, 6 Panxi 7 Road, Jiangbei District, Chongqing 400021, China

## Abstract

**Background:**

Period genes are important core clock genes, including *PER1*, *PER2*, and *PER3*. A number of studies have demonstrated that the abnormal expression of the *PER* gene family of clock genes is associated with the survival and prognosis of patients with cancer; however, the sample sizes included in the majority of these studies were small, and the reported results were inconsistent. This study was the first to collect the relevant publications to systematically evaluate the value of the expression of the *PER* gene family in the prediction of survival and prognosis of human tumors.

**Methods:**

The PubMed, Cochrane Library, Embase, and Web of Science databases were searched systematically, and a meta-analysis was performed.

**Results:**

A total of 12 eligible publications met the inclusion criteria for the meta-analysis, including 1,369 patients and 9 different types of cancer. The pooled hazard ratio for overall survival indicated that the overall survival of patients in the high PER1, PER2, and PER3 protein expression group was significantly higher than that in the low-expression group, respectively. The sensitivity analysis revealed that the result was stable and reliable. The association between *PER1* and *PER3* mRNA expression levels and cancer prognosis was not meta-analyzed as the number of experimental studies was <3. There was no significant association between the expression of *PER2* mRNA and the overall survival of patients with cancer.

**Conclusion:**

PER1, PER2, and PER3 protein expression levels can be used as novel potential biomarkers for predicting cancer prognosis.

## 1. Introduction

The circadian clock is an endogenous adaptive regulatory system formed by the long-term evolution of organisms to adapt to the living environment of the earth's rotation [1]. The circadian clock is primarily composed and regulated by the circadian clock genes, which exist in almost all cells of the human body [2]. Current research has reported that the clock genes regulate ~43% of protein-coding genes in the genomes of mammals [3], thus regulating numerous complex life activities in the body. The period (*PER*)1/2/3 genes are core clock genes and form part of the *PER* gene family [4, 5]. Previous studies have suggested that the *PER* gene family plays a role in the regulation of cell physiological processes, such as the cell cycle [6], DNA damage response [6, 7], cell proliferation, and apoptosis [8]. Abnormal expression of the *PER* genes has been demonstrated to be associated with the occurrence and development of cancer [9–12].

Previous studies have revealed that *PER1-3* expression levels are significantly changed in gastric cancer, colorectal cancer, head and neck squamous cell carcinoma, non-small cell lung cancer, pancreatic cancer, prostate cancer, chronic lymphocytic leukemia, melanoma, hepatocellular carcinoma, breast cancer, and so on [13–22]. The abnormal expression of the *PER* genes is associated with the survival time of patients with cancer [23]. However, the sample size of the majority of the published studies is small, and the reported results are discrete and inconsistent [13–15, 24, 25]. To the best of our knowledge, the present study is the first to collect the relevant literature currently published and to construct a meta-analysis model that was aimed at systematically evaluating the value of the expression of the *PER* gene family in predicting the clinical prognosis of patients with cancer.

## 2. Materials and Methods

### 2.1. Search Strategy

This report has been structured on the basis of PRISMA [26]. The PubMed, Cochrane Library, Embase, and Web of Science databases were independently searched, and all literature published before April 2, 2019, was searched. The following keywords were used in the search process: (“*PER1*” or “*PER2*” or “*PER3*” or “period1” or “period2” or “period3”) and (“cancer” or “carcinoma” or “tumor” or “neoplasm” or “tumour”) and (“survival” or “prognosis” or “outcome” or “predict”). See Table S1 in the Supplementary Material for the literature search strategy. The reference lists of the retrieved articles were also screened manually to obtain any missing literature that met the requirements.

### 2.2. Inclusion and Exclusion Criteria

**Figure 1:**
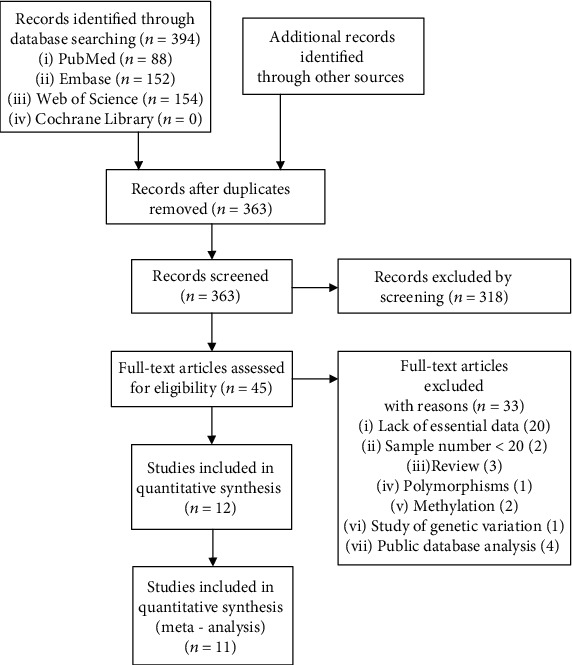
Flow diagram of the process of selection of relevant studies analyzing the prognostic value of period family in cancer.

The study eligibility criteria were established by two authors (Yang and Deng). Any discrepancies were resolved by consensus or determined by a third party (Zheng). The present study focused on the association between the expression of mRNA and protein in the *PER* gene family and overall survival (OS; OS was defined as the time from diagnosis to mortality from any cause). All studies met the following inclusion criteria: (1) cohort or case-control studies, (2) evaluated the association between the expression of the *PER* gene family and cancer prognosis, (3) provided a diagnosis of cancer via pathological methods, (4) provided sufficient information for calculating the hazard ratio (HR) with 95% confidence interval (CI) for OS, (5) the patients were divided into a *PER*-positive group and *PER*-negative group or a high-expression group and low-expression group for the survival analysis, (6) the sample size of the patients was not <20, and (7) published in the English language, and the exclusion criteria are the following: (1) reviews, comments, letters, or case reports; (2) in vitro and animal experimental studies; (3) the same patient samples in different literature; (4) genetic variation of *PER* gene (polymorphism or methylation pattern); and (5) public database analysis. A flow diagram of the article selection process is presented in Figure 1.

### 2.3. Data Extraction and Quality Assessment

The literature was independently screened and extracted according to the aforementioned inclusion and exclusion criteria. The primary prognostic outcome index was OS, and the following data were extracted: the first author, year of publication, geographical area, cancer type, preoperative and postoperative treatment information, *PER* gene expression level, detection method, follow-up time, cut-off value, multivariate or univariate analysis model, age of patients, number of patients, HR value, and 95% CI. HRs and 95% CIs of OS were directly extracted if they were provided in the articles. Or HRs and 95% CIs of OS were estimated via Kaplan-Meier survival curves using the software Engauge Digitizer (version 4.1; http://markummitchell.github.io/engauge-digitizer) according to the method reported by Tierney et al. [27]. The quality of included studies was independently evaluated according to the Newcastle-Ottawa scale (NOS) criteria [28]. The quality scores ranged from 0 (lowest) to 9 (highest); studies with a score > 6 were considered high quality, and low-quality studies were excluded. Any disagreements were resolved by consensus or determined by a third party.

### 2.4. Statistical Analysis

In the present meta-analysis, the association between *PER* expression and cancer prognosis was assessed using pooled HRs and 95% CIs of OS. A HR > 1 indicated poor prognosis in patients with high *PER* expression. In contrast, a HR < 1 indicated improved prognosis in patients with high *PER* expression. *χ*^2^ test and *I*^2^ test were used to identify statistical heterogeneity. If *p* ≥ 0.1 and *I*^2^ < 50%, the results indicated that there was homogeneity among the research results, and the fixed-effect model was used for the meta-analysis. If *p* < 0.1 and *I*^2^ ≥ 50%, the results indicated that there was heterogeneity among the research results, and a random effects model was used. The effectiveness and reliability of the meta-analysis were assessed using a sensitivity analysis, which evaluated the impact of a single study on the results of the overall analysis. The publication bias was evaluated using a funnel diagram or Begg's test. All statistical analyses were performed using Stata software (version 14.0; Stata Corporation). In addition to the heterogeneity test, *p* < 0.05 was considered to indicate a statistically significant result.

## 3. Results

### 3.1. Literature Search Results

A total of 394 articles were searched from the database according to the search strategy. Of these, 12 articles were considered eligible according to the inclusion and exclusion criteria after screening [13, 16, 17, 29–37], which included a total of 1,369 cases and 9 types of cancer. Two articles evaluated gastric cancer (GC) [13, 34], one article evaluated oral squamous cell carcinoma (OSCC) [29], one article evaluated non-small cell lung cancer (NSCLC) [16], two articles evaluated colon cancer (CC) [30, 31], one article evaluated pancreatic ductal adenocarcinoma (PDA) [17], two articles evaluated colorectal cancer (CRC) [35, 37], one article evaluated breast cancer (BC) [32], one article evaluated glioblastoma (GBM) [33], and one article evaluated head and neck squamous cell carcinoma (HNSCC) [36]. The characteristics of the eligible studies are presented in Table 1. No new research was identified in the references lists. The study on the association between only one gene (*PER1*, *PER2*, or *PER3*) mRNA expression level or protein expression level and OS in one literature was regarded as an independent study. However, certain publications reported the associations between two or three *PER* family genes and OS [13, 16, 34, 36]. Therefore, a total of 12 articles with 20 independent studies were included in the present meta-analysis. A total of 70% (14/20) of the studies demonstrated that the expression level of *PERs* was significantly associated with the survival and prognosis of patients with cancer. The remaining 30% (6/20) of the studies suggested that there was no significant association between *PER* expression and the survival and prognosis of patients with cancer. The *PER1*, *PER2*, and *PER3* mRNA expression and protein expression level were determined in cancerous tissues. Cut-off values were used to divide the expression of *PER1-3* into high and low levels (or positive and negative groups). Regarding detection method, the protein expression level of PER1-3 was evaluated by immunohistochemistry (IHC). The cut-off value for the PER1-3 protein expression was based on the staining intensity score and percentage of positive cells (SP). The mRNA expression level of *PER1-3* was evaluated by real-time quantitative reverse transcriptase-polymerase chain reaction (qRT-PCR). The cut-off value for the *PER1-3* mRNA expression was based on the median or mean value. Expression (+) is defined as the positive expression rate of the *PER* family, indicating the proportion of high-expression specimens in the total specimens according to the cut-off point. Of the 12 publications included, 6 (with a total of 11 studies) assessed the PER protein expression level. Among them, there were 4 studies with a total of 476 cases that assessed PER1 protein expression in the prognosis of cancer. There were 4 studies with a total of 639 cases that assessed PER2 protein expression in the prognosis of cancer and 3 studies with a total of 393 cases that assessed PER3 protein expression in the prognosis of cancer. There were 6 publications (with a total of 9 studies) that assessed the *PER* mRNA expression level. Among them, there were 2 studies with a total of 89 cases that assessed *PER1* mRNA expression in the prognosis of cancer. There were 6 studies with a total of 427 cases that assessed *PER2* mRNA expression in the prognosis of cancer and only 1 study with a total of 29 cases that assessed *PER3* mRNA expression in the prognosis of cancer. The year of publication of the 12 articles included was centralized between 2011 and 2018, and the sample sizes were between 23 and 246. There were 3 studies performed in Europe, 1 in Japan, 2 in Taiwan, and 6 in China. NOS scores were ≥6 in all of the included studies. See Table S2 in the Supplementary Material for the Newcastle-Ottawa scale to assess the quality of the included studies.

### 3.2. Prognostic Value of PER1 for OS

**Figure 2:**
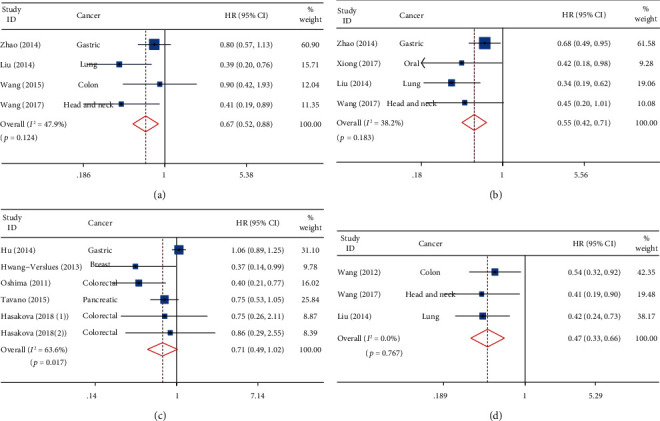
Forest plot of the HR for the relationship between *PER* family expression and OS. (a) Pooled HR for PER1 protein expression and OS. The detection method of PER1 protein level was IHC. (b) Pooled HR for PER2 protein expression and OS. The detection method of PER2 protein level was IHC. (c) Pooled HR for *PER2* mRNA expression and OS. The detection method of *PER2* mRNA expression was qRT-PCR. (d) Pooled HR for PER3 protein expression and OS. The detection method of PER3 protein level was IHC.

A total of 4 studies reported the association between the protein expression level of PER1 and cancer prognosis. The fixed-effect model was used due to the heterogeneity (*I*^2^, 47.9%; *p* = 0.124). The results revealed that the expression of the PER1 protein was significantly associated with OS (HR, 0.67; 95% CI, 0.52-0.88; *p* = 0.003) (Figure 2(a)). Patients with a high expression of the PER1 protein had improved prognosis. Only 2 studies reported the association between *PER1* mRNA expression and OS. Among them, Pluquet et al. reported that patients with high expression of *PER1* mRNA in gliomas had significantly higher OS [33]. Hu et al. demonstrated that there was no significant association between the expression of *PER1* mRNA and OS in patients with gastric cancer [34].

### 3.3. Prognostic Value of PER2 for OS

A total of 4 studies reported the association between the expression of the PER2 protein and OS. Due to the heterogeneity (*I*^2^, 38.2%; *p* = 0.183), the fixed-effect model was used. The results revealed that the expression of the PER2 protein was significantly associated with OS (HR, 0.55; 95% CI, 0.42-0.71; *p* < 0.001) (Figure 2(b)); the high expression of the PER2 protein demonstrated an improved prognosis. A total of 6 studies reported the association between the expression of *PER2* mRNA and cancer prognosis. The random-effect analysis, which was used as the heterogeneity, was significant (*I*^2^, 63.6%; *p* = 0.017). The results suggested that there was no significant association between the expression of *PER2* mRNA and OS in patients with cancer (HR, 0.92; 95% CI, 0.80-1.06; *p* = 0.247; Figure 2(c)).

### 3.4. Prognostic Value of PER3 for OS

A total of 3 studies reported the association between the expression of the PER3 protein and OS; no significant heterogeneity was observed (*I*^2^, 0.0%; *p* = 0.767); therefore, the fixed-effect model was used for the meta-analysis. The results revealed that the expression of the PER3 protein was significantly associated with OS (HR, 0.47; 95% CI, 0.33-0.66; *p* < 0.001; Figure 2(d)). The patients with high expression of the PER3 protein had an improved prognosis. Only 1 study reported an association between the expression of *PER3* mRNA and OS. Hu et al. suggested that the expression of *PER3* mRNA was significantly associated with OS in patients with gastric cancer [34].

### 3.5. Sensitivity Analysis

**Figure 3:**
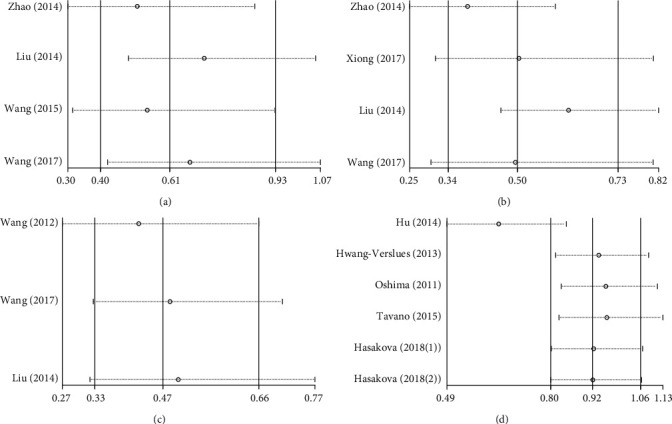
Sensitivity analysis of OS. (a) Sensitivity analysis for PER1 protein expression and OS. (b) Sensitivity analysis for PER2 protein expression and OS. (c) Sensitivity analysis for PER3 protein expression and OS. (d) Sensitivity analysis for *PER2* mRNA expression and OS.

In order to investigate the stability of the pooled HRs for the PER1-3 proteins, a sensitivity analysis was performed. After removing one study at a time, there was no significant change in the total meta-analysis results of the remaining studies (Figures 3(a)–3(c)), which indicated that the results of the 3 studies are statistically stable and reliable.

In order to investigate the stability of the pooled HRs for *PER2* mRNA, a sensitivity analysis was performed. After removing the study by Hu et al. [34], the meta-analysis demonstrated that the results changed significantly from the original results that did not have statistical significance (HR, 0.92; 95% CI, 0.80-1.06; *p* = 0.247) to the results with statistical significance (HR, 0.63; 95% CI, 0.47-0.85; *p* = 0.002). The heterogeneity changed from the original large heterogeneity (*I*^2^, 63.6%; *p* = 0.017) to homogeneity (*I*^2^, 7.6%; *p* = 0.363). However, after removing the study by other authors at a time, there was no significant change in the total meta-analysis results. These results demonstrate that the great heterogeneity in the present study is caused by the difference between the research of Hu et al. and the quality of the other studies (Figure 3(d)).

### 3.6. Publication Bias

**Figure 4:**
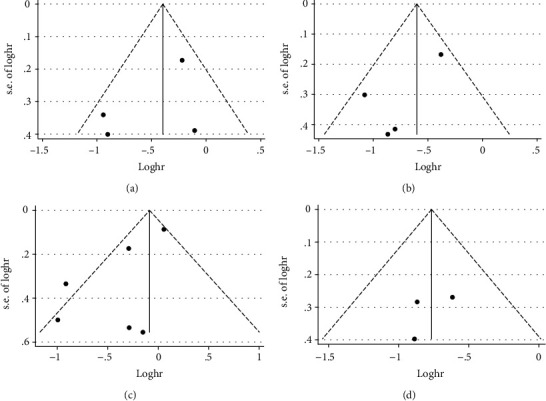
Funnel plot of OS for publication bias. (a) Funnel plot for PER1 protein expression and OS. (b) Funnel plot for PER2 protein expression and OS. (c) Funnel plot for *PER2* mRNA expression and OS. (d) Funnel plot for PER3 protein expression and OS.

**Figure 5:**
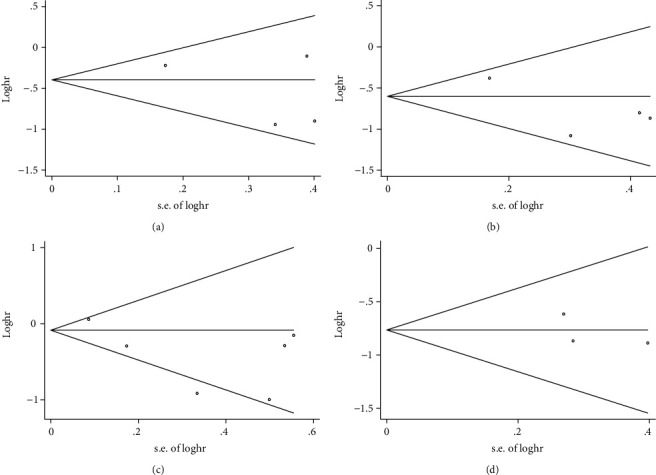
Begg's funnel plot of OS for publication bias. (a) Begg's funnel plot for PER1 protein expression and OS (*p*_Begg's_ = 0.734). (b) Begg's funnel plot for PER2 protein expression and OS (*p*_Begg's_ = 0.734). (c) Begg's funnel plot for *PER2* mRNA expression and OS (*p*_Begg's_ = 1.000). (d) Begg's funnel plot for PER3 protein expression and OS (*p*_Begg's_ = 1.000).

In order to investigate the publication bias of the present study, funnel plots and Begg's test analyses were performed (Figures 4(a)–4(d) and 5(a)–5(d)). The results demonstrated that there was no significant publication bias in the present study.

## 4. Discussion

In the present systematic review and meta-analysis, all the published literature was collected in order to investigate the potential role of the expression level of circadian clock genes in the *PER* family in predicting the survival and prognosis of human cancer. From the included publications, it was revealed that in the currently published studies assessing the associations between *PER* gene family expression and cancer prognosis, the primary indicator was OS. Only Wang et al. reported an association between *PER1* expression level and disease-free survival (DFS) [30]. Wang et al. [31] reported an association between the *PER3* expression level and DFS, while Wang et al. [36] reported an association between the expression level of *PER1-3* and progression-free and recurrence-free survival. Therefore, the present study selected the most representative indicator of OS to pursue the research. The research results demonstrated that the protein expression levels of PER1, PER2, and PER3 were significantly associated with the survival and prognosis of patients with cancer. However, there was no significant association between the expression of *PER2* mRNA and the survival and prognosis of patients with cancer. *PER1* mRNA and *PER3* mRNA were not included in the present meta-analysis as there were fewer than three research projects which were included in the study.

The present study analyzed the association between the expression of the *PER* family of clock genes and cancer prognosis for the first time via systematic review and meta-analysis. The change of gene mRNA expression level is not necessarily consistent with the change of protein expression level [38, 39]; therefore, the present study analyzed the association between the *PER* gene family mRNA and protein expression level and cancer prognosis. The following results were obtained: patients with cancer that exhibited high-expression levels of the PER1-3 proteins had improved prognosis compared with those with low expression, which suggests that the PER1-3 proteins may be new biomarkers for predicting prognosis in patients with cancer. However, there are few researches exploring the association between *PER1-3* mRNA expression levels and cancer prognosis; thus, additional original studies with consistent research standards and large sample sizes are needed for further evaluation. Although we have made a comprehensive analysis to the included literature, certain limitations still remain: First, the present study included those literature that can be searched in the PubMed, Cochrane Library, Embase, and Web of Science databases. It should be acknowledged that literature from other databases may have been missed. Secondly, the positive results are often easier to publish than the negative results when regarding the acquisition of data, and thus, the existence of publication bias may cause some errors in the pooled results [40]. Thirdly, the HRs from part of the survival data were extracted using the Kaplan-Meier method. Compared with the data extracted directly from the original articles, there may be some errors in this part of the data, which may also affect the pooled results.

## 5. Conclusion

The present study identified for the first time that expression of the *PER* family of clock genes is closely associated with the OS of patients with cancer via the systematic review and meta-analysis. Patients with high expression of *PER* family protein have better prognosis than those with low expression. The expression level of the *PER* gene family may be used as novel potential biomarkers to predict cancer prognosis. This provides a basis and the theoretical support required for studies regarding the role of the *PER* gene family in cancer in the future.

## Figures and Tables

**Table 1:** Main characteristics of the studies included in the meta-analysis.

Study (publish year)	Country	Cancer type	Gene type	Sample size	Detection method	Follow-up (months)	Expression (+) (%)	Cut-off	Age	Stage	NOS scores
Zhao (2014) [13]	China	GC	*PER1*	246	IHC	25 (1-161)	(103/143) 41.87%	SP > 4	57.1 (23–79)	I-IV	8
Zhao (2014) [13]	China	GC	*PER2*	246	IHC	25 (1-161)	(86/160) 34.96%	SP > 4	57.1 (23–79)	I-IV	8
Xiong (2017) [29]	China	OSCC	*PER2*	40	IHC	>60	(16/24) 40%	SP ≥ 2	N/A	I-IV	8
Liu (2014) [16]	China	NSCLC	*PER1*	130	IHC	>60	(86/44) 66.15%	SP > 4	61 (38–79)	I-III	8
Liu (2014) [16]	China	NSCLC	*PER2*	130	IHC	>60	(77/53) 59.23%	SP > 4	61 (38–79)	I-III	8
Liu (2014) [16]	China	NSCLC	*PER3*	130	IHC	>60	(82/48) 63.08%	SP > 4	61 (38–79)	I-III	8
Wang (2015) [30]	China	CC	*PER1*	203	IHC	61 (9-89)	(182/21) 89.66%	SP ≥ 2	68 (22–95)	I-IV	8
Wang (2012) [31]	China	CC	*PER3*	203	IHC	61 (9-89)	(167/36) 82.27%	SP ≥ 2	65 (22–95)	I-IV	8
Tavano (2015) [17]	Italy	PDA	*PER2*	34	qRT-PCR	25.2	N/A	N/A	68.5 (51–74)	IIA-III	8
Oshima 2011 [37]	Japan	CRC	*PER2*	202	qRT-PCR	>60	(101/101) 50%	Median value	66	N/A	8
Hwang-Verslues (2013) [32]	Taiwan	BC	*PER2*	101	qRT-PCR	0-120	(50/51) 49.50%	N/A	53.8 ± 12.6	N/A	6
Pluquet (2013) [33]	France	GBM	*PER1*	60	qRT-PCR	>80	(31/29) 51.67%	Average value	N/A	N/A	7
Hu (2014) [34]	Taiwan	GC	*PER1*	29	qRT-PCR	60	N/A	N/A	69.76 (51–81)	I-IV	7
Hu (2014) [34]	Taiwan	GC	*PER2*	29	qRT-PCR	60	N/A	N/A	69.76 (51–81)	I-IV	7
Hu (2014) [34]	Taiwan	GC	*PER3*	29	qRT-PCR	60	N/A	N/A	69.76 (51–81)	I-IV	7
Wang (2017) [36]	China	HNSCC	*PER1*	60	IHC	0-60	(29/31) 48.33%	SP ≥ 2	59 (41–84)	I-IV	9
Wang (2017) [36]	China	HNSCC	*PER2*	60	IHC	0-60	(27/33) 45%	SP ≥ 2	59 (41–84)	I-IV	9
Wang (2017) [36]	China	HNSCC	*PER3*	60	IHC	0-60	(29/31) 48.33%	SP ≥ 2	59 (41–84)	I-IV	9
Hasakova (2018) [35]	Slovak Republic	CRC	*PER2*	38	qRT-PCR	0-60	(19/19) 50%	Median value	69 ± 12	I-IV	6
Hasakova (2018) [35]	Slovak Republic	CRC	*PER2*	23	qRT-PCR	0-60	(11/12) 47.83%	Median value	69 ± 12	I-IV	6

GC: gastric cancer; CRC: colorectal cancer; CC: colon cancer; PDA: pancreatic ductal adenocarcinoma; BC: breast cancer; GBM: glioblastoma; NSCLC: non-small cell lung cancer; OSCC: oral squamous cell carcinoma; HNSCC: head and neck squamous cell carcinoma; IHC: immunohistochemistry; qRT-PCR: real-time quantitative reverse transcriptase-polymerase chain reaction; SP: staining intensity score and percentage of positive cells; NA: not available.

## Data Availability

The data used to support the findings of this study are available from the included published articles and the corresponding author upon request.

## Supplementary Materials

Supplementary materials Table S1: literature search strategy. (Supplementary Materials). Table S2: Newcastle-Ottawa scale to assess the quality of the included studies.
