# Supplementary material for: Period Family of Clock Genes as Novel Predictors of Survival in Human Cancer: A Systematic Review and Meta-Analysis
Source: Dis Markers. 2020 Aug 10;2020:6486238. doi: 10.1155/2020/6486238 (PMC7436287; doi:10.1155/2020/6486238)
Supplement: Supplementary materials — Table S1: literature search strategy. (Supplementary Materials). Table S2: Newcastle-Ottawa scale to assess the quality of the included studies. [file 6486238.f1.doc]

**Period family of clock genes as novel predictors of survival in human cancer: A systematic review and meta-analysis**

Fan Deng^1^, Kai Yang^1*^ , Gang Zheng^2*^

**Supplementary material**

Table S1: Literature search strategy.

| **Strategy used to search PubMed**  ((((((((PER3) OR Period3)) OR ((PER2) OR Period2)) OR ((PER1) OR Period1))) AND ((((((((cancer) OR tumor) OR tumour) OR carcinoma) OR malignan*) OR neoplasm*)) OR "Neoplasms"[Mesh]))) AND ((((survival) OR prognos*) OR predict) OR outcome) |
| --- |
| **Strategy used to search Embase**  ('neoplasm'/exp OR 'neoplasm' OR cancer OR tumor OR carcinoma) AND (survival OR prognosis OR outcome OR predict) AND (PER1 OR PER2 OR PER3 OR Period1 OR Period2 OR Period3) |
| **Strategy used to search Web of Science**  #1 TS= (PER1 OR Period1)  #2 TS= (PER2 OR Period2)  #3 TS= (PER3 OR Period3)  #4 TS= (neoplasms OR neoplasm* OR cancer OR tumor OR tumour OR carcinoma OR malignan*)  #5 TS= (survival OR prognos* OR outcome OR predict)  #6 TS= #1 OR #2 OR #3  #7 TS= #4 AND #5 AND #6 |
| **Strategy used to search Cochrane library**  #1 neoplasm*:ME  #2 neoplasm* OR cancer OR tumor OR tumour OR carcinoma OR malignan*  #3 (#1 OR #2)  #4 PER1 OR PER2 OR PER3 OR Period1 OR Period2 OR Period3  #5 survival OR prognos* OR outcome OR predict  #6 (#3 AND #4 AND #5) |

Table S2: NewCastle Ottawa scale to assess the quality of the included studies.

| **Study** | **Selection** | | | | **Comparability** | **Outcome** | | |  |
| --- | --- | --- | --- | --- | --- | --- | --- | --- | --- |
|  | **Represent-ative of cases** | **Selection of controls** | **Ascertain-ment of exposure** | **Outcome** | **Comparability of the design or analysis** | **Assessment of outcome** | **Adequate follow-up time** | **Adequacy of follow up** | **NOS scores** |
| **Zhao 2014 ^[13]^** | **1** | **1** | **1** | **1** | **2** | **1** | **1** | **0** | **8** |
| **Xiong 2017 ^[29]^** | **1** | **1** | **1** | **1** | **2** | **1** | **1** | **0** | **8** |
| **Liu 2014 ^[16]^** | **1** | **1** | **1** | **1** | **2** | **1** | **1** | **0** | **8** |
| **Wang 2015 ^[30]^** | **1** | **1** | **1** | **1** | **2** | **0** | **1** | **1** | **8** |
| **Wang 2012 ^[31]^** | **1** | **1** | **1** | **1** | **2** | **0** | **1** | **1** | **8** |
| **Tavano 2015 ^[17]^** | **1** | **1** | **1** | **1** | **2** | **1** | **1** | **0** | **8** |
| **Oshima 2011 ^[37]^** | **1** | **1** | **1** | **1** | **2** | **1** | **1** | **0** | **8** |
| **Hwang-Verslues 2013 ^[29]^** | **1** | **1** | **1** | **1** | **2** | **0** | **0** | **0** | **6** |
| **Pluquet 2013 ^[33]^** | **1** | **1** | **1** | **1** | **2** | **1** | **0** | **0** | **7** |
| **Hu 2014 ^[34]^** | **1** | **1** | **1** | **1** | **2** | **0** | **1** | **0** | **7** |
| **Wang 2017 ^[36]^** | **1** | **1** | **1** | **1** | **2** | **1** | **1** | **1** | **9** |
| **Hasakova 2018 ^[35]^** | **1** | **1** | **1** | **1** | **2** | **0** | **0** | **0** | **6** |
